# Increase in testosterone levels is related to a lower risk of conversion of prediabetes to manifest diabetes in prediabetic males

**DOI:** 10.1007/s00508-021-01903-1

**Published:** 2021-07-05

**Authors:** Michael Leutner, Caspar Matzhold, Luise Bellach, Evelyne Wohlschläger-Krenn, Robert Winker, Sonja Nistler, Georg Endler, Stefan Thurner, Peter Klimek, Alexandra Kautzky-Willer

**Affiliations:** 1 Department of Internal Medicine III, Clinical Division of Endocrinology and Metabolism, Unit of Gender Medicine, Medical University of Vienna, Währinger Gürtel 18–20, 1090 Vienna, Austria; 2 Section for Science of Complex Systems, CeMSIIS, Medical University of Vienna, Spitalgasse 23, 1090 Vienna, Austria; 3 Complexity Science Hub Vienna, Josefstädter Straße 39, 1080 Vienna, Austria; 4 Health and Prevention Center KFA, Löblichgasse 14, 1090 Vienna, Austria; 5 Santa Fe Institute, 1399 Hyde Park Road, Santa Fe, NM 85701, USA; 6 IIASA, Schlossplatz 1, 2361 Laxenburg, Austria; 7 Gender Institute, 3571 Gars am Kamp, Austria

**Keywords:** Prediabetes, Testosterone, Insulin resistance, Fatty liver, Sex-specific differences

## Abstract

**Background:**

Testosterone plays an important role in the regulation of glucose metabolism. While earlier studies have shown that it has a protective effect in males, unfavorable effects of testosterone on glucose metabolism have been reported in females; however, whether there is a sex-specific relationship between testosterone and glucose metabolism in patients with prediabetes has not been investigated in detail hitherto.

**Methods:**

This cross-sectional analysis investigated 423 males and 287 females with diagnosed prediabetes. Detailed assessment of their metabolic profiles was performed, including a 2‑h oral glucose tolerance test (OGTT), HbA1c levels, calculation of insulin resistance with homeostatic model assessment for insulin resistance (HOMA-IR), assessment of lipid metabolism, anthropometric parameters and the fatty liver index (FLI). By using Spearman’s correlation test, we investigated the sex-specific relationship between testosterone and metabolism in the prediabetic individuals.

**Results:**

In the present study, prediabetic females (mean age 58.6 years, confidence interval [CI: 57.6 y; 59.5 y]) were characterized by lower fasting plasma glucose levels (104.2 mg/dl [CI: 103.0 mg/dl; 105.4 mg/dl] vs. 106.9 mg/dl [CI: 106.0 mg/dl; 107.8 mg/dl]) and a lower FLI (49.5 [CI: 45.7; 53.2] vs. 58.8 [CI: 55.8; 61.8]), but presented with a higher risk of developing manifest type 2 diabetes in the next 10 years (FINDRISK score: 17.6 [CI: 17.1; 18.1] vs. 16.1 [CI: 15.7; 16.5]) when compared to prediabetic males (mean age: 58.04 years [CI: 57.0 y; 59.1 y]). Testosterone was negatively related to insulin resistance (HOMA-IR: Spearman’s ρ: −0.33, *p* < 0.01), 2‑h stimulated glucose levels during the OGTT (ρ = −0.18, *p* < 0.01), HbA1c levels (ρ = −0.13, *p* < 0.05), FLI and BMI in prediabetic males; however, no relationship between testosterone and metabolic parameters could be found in prediabetic females.

**Conclusion:**

The increase of testosterone levels in males was related to a more favorable glucose metabolism, including lower HbA1c, lower stimulated glucose levels and higher insulin sensitivity; however, in prediabetic females, testosterone was not related to glucose metabolism.

## Introduction

In recent years, the prevalence of type 2 diabetes mellitus (T2DM) has been steadily increasing and is predicted to rise even further worldwide by 2045. Latest numbers from the International Diabetes Federation demonstrate that 9.3% of the world’s population have T2DM [1]. Physiologically, sex hormones are involved in the regulation of glucose metabolism and are essential for maintenance of glucose homeostasis [2]. In men, testosterone is linked to insulin sensitivity and glycemic control [3]. Accordingly, in males there is a correlation between testosterone deficiency and the prevalence of prediabetes [4] and insulin resistance [5], meaning testosterone has favorable effects on glucose metabolism in males. Furthermore, low testosterone and sex hormone binding globulin (SHBG) levels in men can be linked to increased visceral fat content [6]. Conversely, higher testosterone levels and testosterone replacement therapy show beneficial effects on glucose homeostasis in men [7]; however, this does not seem to apply to women, since high testosterone blood levels in women are associated with T2DM [2, 3] and have been linked to diseases such as polycystic ovary syndrome [8]. Interestingly, there are sex-specific differences in the diagnosis of disturbances in glucose metabolism between men and women. Men more often suffer from increased fasting plasma glucose levels (FPG), whereas women more often suffer from impaired glucose tolerance, which is diagnosed with the oral glucose tolerance test (OGTT). The pathophysiological mechanisms behind these sex-specific differences are not entirely clear to date; however, one of the main reasons for the higher occurrence of increased FPG levels in men may be the elevated hepatic glucose output as well as impaired early insulin secretion. On the other hand, women more often suffer from impaired glucose tolerance (IGT) and an explanation for these sex-specific differences may be the slower duration of gastric emptying and intestinal glucose absorption in women [9]; however, especially the influence of sex hormones on disturbances of glucose metabolism in prediabetic patients is, overall, an unexplored area. Hence this study aimed to investigate the relationship between testosterone and glucose metabolism in prediabetic patients in a sex-specific manner.

## Methods

### Study design and methods

The cross-sectional study at hand (ethics approval from the Medical University of Vienna, 1089/2017) is a retrospective data analysis with an explorative character of first visits to the prediabetes clinic at “Health and Prevention Center KFA” in Vienna from October 2015 to September 2017. As part of a standard medical check-up, patients underwent a thorough physical examination, electrocardiogram, extensive blood analysis and assessment of vital parameters. Patients were classified as prediabetic patients according to the guidelines of the American Diabetes Association (ADA) if HbA1c was ≥ 5.7% and < 6.5% and/or if they had fasting blood glucose levels of ≥ 100 mg/dl and < 126 mg/dl and/or ≥ 140 mg/dl and < 200 mg/dl in a 2‑h OGTT. All patients gave written informed consent. Patients suffering from chronic or acute infections, severe endocrine diseases (such as Cushing’s disease), known hepatitis infection, HIV, present malignant diseases, systemic cortisone treatment, or testosterone substitution were excluded from the analysis.

### Calculations

We calculated the homeostasis model assessment insulin resistance score (HOMA-IR) using basal insulin and glucose levels (HOMA-IR = Ins × Gluc/405), the fatty liver index (FLI) and the Finnish diabetes risk score (FINDRISK score) [10, 11].

### Statistical analysis

Descriptive data analysis was performed for all parameters. Continuous variables were summarized by mean and confidence intervals. Assumption of Gaussian distribution of parameters was decided by visual assessment of histograms and quantile-quantile plots (QQ plots). For normal distributed variables, a two-sided t‑test was used to assess differences between the two groups, and a Mann-Whitney U test was used for non-parametrically distributed variables. Due to the heterogeneous/skewed distribution of the individual variables, the Spearman correlation test was used for all groups to ensure reliable, comparable results. The statistical analysis was performed using the open source statistical analysis software R (version 4.01).

## Results

### Baseline characteristics

Table 1 shows the sex-specific comparison of the baseline characteristics between prediabetic men and women. In this analysis (f: 58.6 [CI: 57.6; 59.5] vs. m: 58.0 [CI: 57.0; 59.1] years), prediabetic males were characterized by lower total cholesterol and HDL cholesterol levels when compared to prediabetic females. The amount of visceral fat in the liver, measured with the fatty liver index, was significantly lower in prediabetic females.

**Table 1:** Baseline characteristics of prediabetic females and males

	Males			Females			
Variable	*N*	Mean	95%CI	*N*	Mean	95%CI	*p*-value
Age (years)	423	58.0	(57.0; 59.1)	287	58.6	(57.6; 59.5)	0.48
BMI (kg/m^2^)	419	28.2	(27.6; 28.7)	284	28.3	(27.7; 28.9)	0.87
Cholesterol (mg/dl)	415	205.6	(201.8; 209.5)	278	218.9	(214.4; 223.4)	< 0.01
HDL cholesterol (mg/dl)	416	56.2	(54.8; 57.7)	279	65.9	(64.0; 67.8)	< 0.01
LDL cholesterol (mg/dl)	413	125.9	(122.4; 129.4)	279	130.8	(126.6; 34.9)	0.08
Triglycerides (mg/dl)	416	115.7	(109.4; 121.9)	279	110.2	(104.6; 115.8)	0.88
Fatty liver Index	294	58.8	(55.8; 61.8)	197	49.5	(45.7; 53.2)	< 0.01
Find risk score	299	16.1	(15.7; 16.5)	196	17.6	(17.1; 18.1)	< 0.01
Glucose (mg/dl)	402	106.9	(106.0; 107.8)	267	104.2	(103.0; 105.4)	< 0.01
Insulin (µU/ml)	279	12.6	(11.6; 13.6)	181	13.1	(12.1; 14.1)	0.07
HOMA-IR	197	3.5	(3.2; 3.8)	134	3.4	(3.1; 3.8)	0.84
Glucose levels (mg/dl)—OGTT 2 h	289	117.4	(113.8; 121.0)	196	119.8	(115.3; 124.3)	0.41
Fasting glucose levels (mg/dl)—OGTT	291	108.5	(107.5; 109.4)	196	106.7	(105.4; 108.0)	0.03
Testosterone (ng/ml)	284	4.5	(4.3; 4.7)	176	0.2	(0.2; 0.2)	< 0.01
Vitamin D (ng/ml)	404	27.2	(26.1–28.2)	270	29.6	(28.1–31.2)	0.02
HbA1c > 5.7%—≤ 6.5%	131	5.9	(5.8; 5.9)	134	5.9	(5.9; 5.9)	0.22
Fasting glucose 100–< 126 mg/dl	252	109.2	(108.0; 110.0)	147	109.0	(108.0; 110.1)	0.53
Glucose levels 140–< 200 mg/dl after 2 h during the OGTT (mg/dl)	69	159.8	(156.2; 163.5)	42	162.6	(157.6; 167.7)	0.42

*BMI* body mass index, *HDL* high-density lipoprotein, *LDL* low-density lipoprotein, *HOMA-IR* homeostatic model assessment for insulin resistance, *OGTT* oral glucose tolerance test

However, while investigating glucose metabolism, we found a significantly higher FINDRISK score in prediabetic females, which means that they are at higher risk of developing manifest diabetes in the next 10 years. In general, prediabetic females presented with lower fasting plasma glucose (FPG); however, no sex-specific differences could be found for the stimulated glucose levels during the OGTT after 2 h, HbA1c levels or the HOMA-IR.

### Correlation analyses of testosterone with metabolic parameters

**Fig. 1:**
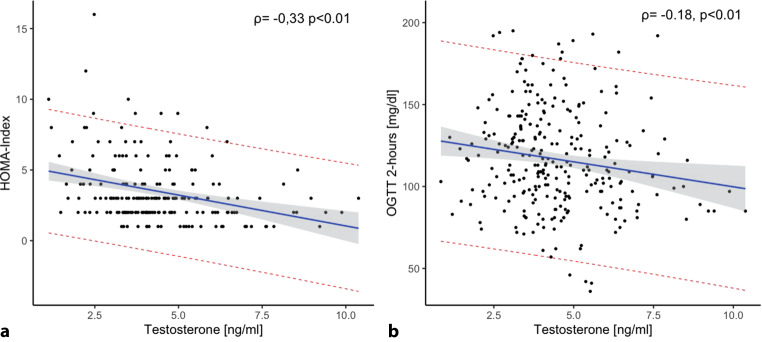
Correlation between testosterone and **a** insulin resistance and **b** stimulated glucose levels in males. The linear relationship is indicated by the *blue regression line* with the corresponding standard error (*gray error bar*). The confidence lines for the predicted values are indicated by a *red dotted line*

In a sex-specific correlation analysis (Table 2) our results show that there is a significant relationship between testosterone and glucose and lipid metabolism and between testosterone and anthropometric parameters in prediabetic males. The higher the levels of testosterone, the lower the levels of HbA1c and the lower the grade of insulin resistance measured with the HOMA index (Fig. 1) were in prediabetic males. Additionally, there was a negative relationship between testosterone and the stimulated glucose levels after 2 h in the OGTT in males (Fig. 1). We could also observe a favorable relationship between testosterone and the visceral fat content in the liver and anthropometric parameters (including FLI and BMI) in prediabetic males. Additionally, there was also a favorable relationship between testosterone and lipid parameters in prediabetic males, including a positive relationship with HDL cholesterol and a negative relationship with triglycerides; however, we could not find a relationship between testosterone and metabolic parameters in prediabetic females.

**Table 2:** Sex-specific correlation analysis of testosterone with metabolic parameters

	Males			Females		
Variable	*N*	ρ	*p*-value	*N*	ρ	*p*-value
Fasting glucose levels (mg/dl)—OGTT	276	−0.06	0.35	174	−0.02	0.80
Glucose levels (mg/dl)—OGTT 2 h	274	−0.18	< 0.01	174	−0.05	0.53
HbA1c (%)	270	−0.13	0.04	170	0.00	0.99
HOMA-IR	195	−0.33	< 0.01	131	−0.08	0.38
Fatty liver index	275	−0.36	< 0.01	171	0.08	0.31
BMI (kg/m^2^)	284	−0.31	< 0.01	174	0.04	0.63
Cholesterol (mg/dl)	282	0.03	0.61	171	0.04	0.62
HDL cholesterol (mg/dl)	283	0.19	< 0.01	172	−0.10	0.18
LDL cholesterol (mg/dl)	281	0.07	0.28	172	0.05	0.52
Triglycerides (mg/dl)	282	−0.18	< 0.01	172	−0.02	0.81

*OGTT* oral glucose tolerance test, *2 h* 2 hours, *HOMA-IR* homeostatic model assessment for insulin resistance, *BMI* body mass index, *HDL* high-density lipoprotein, *LDL* low-density lipoprotein

## Discussion

The present study aimed to examine the sex-specific relationship between testosterone and glucose metabolism in prediabetic men and women. Prediabetic females were characterized by lower FPG levels and a lower FLI but had a higher risk of developing manifest diabetes in the next 10 years when compared to prediabetic men. Interestingly, testosterone seemed to have an overall favorable effect on metabolic profiles in prediabetic males, showing that there was a negative relationship with insulin resistance, HbA1c, stimulated glucose levels during the OGTT, FLI and BMI. Additionally, the increase in testosterone was related to a more favorable lipid profile in prediabetic men; however, in females we could not find a relationship between testosterone and metabolic parameters.

Although there is an abundance of available data showing an effect of testosterone on glucose metabolism in general or, more specifically, T2DM, there is only a limited amount of information on the influence of testosterone in the prediabetic state; however, existing data clearly indicate that prediabetic men show decreased testosterone or SHBG levels [12, 13]. In males, decreased testosterone levels are related to the development of disturbances in glucose metabolism, including increased risk of developing diabetes, and also showed a relationship with increased all-cause and cardiovascular mortality risk [6] as well as greater risk of developing a metabolic syndrome [14]. The significant role of testosterone in glucose metabolism in males is also reflected in the higher likelihood of lowered testosterone levels in T2DM patients compared to non-diabetics [15]. So far, it is not entirely known whether decreased levels of testosterone are the causal factor for metabolic derailment [9]. Nevertheless, one has to keep in mind that testosterone replacement therapy in hypogonadal diabetic males is related to an improvement of insulin sensitivity in patients with T2DM, metabolic syndrome or both [16]. Additionally, in patients receiving an androgen-lowering therapeutic regimen for the treatment of prostate cancer, the amount of visceral fat and insulin resistance increases under this therapy [15]. Furthermore, the detrimental effects androgen deficiency exerts on glucose homeostasis were also demonstrated in animal studies and cell culture experiments in which insulin secretion was impaired on top of testosterone deprivation [17]. Notably, there seems to be a bidirectional relationship between plasma testosterone and the integrity of glucose homeostasis. On the one hand, hyperglycemia suppresses gonadal testosterone production [18], while on the other hand testosterone deficiency leads to decreased muscle mass [19] and hence reduced glucose uptake in muscle [20]. Strikingly, in a prediabetic population, testosterone treatment showed a reduction in waist circumference and body weight, as well as an improvement in HbA1c and FPG—in comparison, 40% of the prediabetic participants in the control group progressed to T2DM [21]. Interestingly, the effect of testosterone on the glucose metabolism seems to play a central role in the development of prediabetes. In a prospective clinical study, Minooee et al. detected that low testosterone levels were related to the development of prediabetes but could not find a relationship between the conversion of prediabetes to T2DM [22]. In the present study, we could show that especially in prediabetic men there is a significant relationship between testosterone and metabolic profiles. Increases in testosterone were related to a lower FLI, better insulin sensitivity, lower HbA1c and lower stimulated glucose levels, which are all markers directly related to the development of T2DM. One possible explanation for improved peripheral insulin sensitivity when testosterone levels are higher is the facilitation of glucose transporter type 4 (GLUT4) translocation to the myocytal membrane and, consequently, greater capacity for peripheral glucose uptake [20]. Furthermore, the protective effect testosterone exerts on peripheral glucose tolerance seems to be robust when correcting for age. Unlike in males, negative effects of testosterone on glucose metabolism have been reported in women [9]. For instance, elevated testosterone levels have been demonstrated in women with diabetes [23]—a constellation also typically present in polycystic ovary syndrome [24–26]. The role of testosterone in the prediabetic state in females is, overall, an unexplored area. Based on earlier results [9, 23], expectation of a negative effect of testosterone on the glucose metabolism in prediabetic females was high; however, in the present study we could not find a relationship between testosterone and insulin resistance, HbA1c, stimulated glucose levels during the OGTT or under fasting conditions and FLI or anthropometric parameters in prediabetic female subjects.

The role of testosterone in the sex-specific diagnostic differences in the disturbances of glucose metabolism is particularly interesting and our results provide more detailed knowledge on this topic. In general, males suffer more often from impaired fasting glucose levels driven by increased hepatic glucose output and dysfunctional early insulin secretion, whereas in females a higher occurrence of impaired glucose tolerance (IGT), mediated via peripheral insulin resistance, has been observed [9]. Interestingly, our results showing that there is a negative relationship between testosterone and stimulated glucose levels in prediabetic males demonstrate that testosterone could be an additional reason for the lower occurrence of IGT in males. As a possible mechanism, Navarro et al. showed that testosterone amplifies the effect of glucagon-like peptide 1 (GLP-1) and enhances beta-cell function [27].

Finally, our results showing that testosterone seems to have favorable effects on the lipid profile in males are in line with earlier studies [6, 21].

Our study has limitations which have to be reported. Firstly, the present study has a cross-sectional study design and thus, we assessed correlations but not causal relationships. Secondly, we did not measure the levels of estrogen as well as the visceral fat content in the liver using gold standard methods such as magnetic resonance spectroscopy or ultrasound. Furthermore, we only assessed bivariate correlations between testosterone and metabolic parameters and therefore cannot rule out that the correlation in females is masked by a correlation with unobserved confounders; however, we noted that both males and females had comparable distributions of age and BMI. We had a smaller sample of females than males, which renders significant results in females less likely; however, males not only had lower *p*-values but also had slightly higher correlation coefficients.

In conclusion, our results show that there is a weak favorable relationship between testosterone and both glucose and lipid metabolism as well as with the visceral fat content in the liver, measured by FLI in prediabetic males. Interestingly, testosterone was not related to the metabolic profile in prediabetic females. Large prospective clinical studies are needed in order to investigate the sex-specific role of testosterone on metabolic profiles in more detail.
